# Insights on Embolic Protection, Repositioning, and Stroke: A Subanalysis of the RESPOND Study

**DOI:** 10.1155/2020/3070427

**Published:** 2020-05-17

**Authors:** Julia Seeger, Volkmar Falk, David Hildick-Smith, Sabine Bleiziffer, Daniel J. Blackman, Mohamed Abdel-Wahab, Dominic J. Allocco, Ian T. Meredith, Jochen Wöhrle, Nicolas M. Van Mieghem

**Affiliations:** ^1^Internal Medicine II, University Hospital of Ulm, Ulm, Germany; ^2^Department of Cardiology and Intensive Care, Medical Campus Lake Constrance, Klinikum Friedrichshafen, Friedrichshafen, Germany; ^3^Department of Cardiothoracic and Vascular Surgery, German Heartcenter Berlin, Berlin, Germany; ^4^Department of Cardiothoracic Surgery, Charite Universitätsmedizin Berlin, Berlin, Germany; ^5^German Center of Cardiovascular Research, Partner Site Berlin, Berlin, Germany; ^6^Department of Health Science and Technology, Swiss Federal Institute of Technology, Zürich, Switzerland; ^7^Sussex Cardiac Centre, Brighton and Sussex University Hospitals, Eastern Road, Brighton, BN2 5BE, UK; ^8^Clinic for Thoracic and Cardiovascular Surgery, Herz-und Diabeteszentrum NRW, Ruhr-University Bochum, Bad Oeynhausen, Germany; ^9^Leeds Teaching Hospitals NHS Trust, Great George Street, Leeds, UK; ^10^Department of Structural Heart Disease/Cardiology, Heart Center Leipzig at University of Leipzig, Leipzig, Germany; ^11^Boston Scientific Corporation, 300 Boston Scientific Way, Marlborough, MA 01752, USA; ^12^Erasmus University Medical Center, Office Bd 171—'s Gravendijkwal 230 3015 CE, Rotterdam, Netherlands

## Abstract

RESPOND is a prospective, single-arm study enrolling 1014 transcatheter aortic valve replacement (TAVR) patients. The objective of this analysis is to assess the impact of cerebral embolic protection (CEP) devices and prosthetic valve repositioning on the risk of neurologic complications in patients treated with the fully repositionable Lotus Valve in the RESPOND postmarket study. Valve repositioning and CEP use were at the operators' discretion. Stroke events were adjudicated by an independent medical reviewer. This analysis assessed the baseline differences among patients according to CEP use and valve repositioning and evaluated the neurological complications at 72 hours after TAVR, hospital discharge, and 30-day follow-up. A multivariate analysis was performed to identify the potential predictors of stroke. Of the 996 patients implanted with the Lotus Valve (mean age: 80.8 years, 50.8% female, STS score 6.0 ± 6.9), 92 cases (9.2%) used CEP. The overall rate of acute stroke/transient ischemic attack (TIA) was 3.0% at 72 hours after TAVR. The 72-hour stroke/TIA rate was 1.1% in patients who had CEP and 3.2% in those who did not. Use of CEP was associated with a 2.1% absolute reduction in the risk of acute neurological events (relative risk reduction: 65.6%), although the difference was not statistically significant (*p*=0.51). Repositioning of the Lotus Valve occurred in 313/996 procedures (31.4%). The 72-hour rate of stroke/TIA was similar in patients who had valve repositioning (2.9%) compared with those who did not (3.1%; *p*=0.86). The selective use of a CEP device in the RESPOND study was associated with a nonsignificantly lower risk for stroke within 72 hours. The use of the repositioning feature of the Lotus Valve did not increase the stroke risk.

## 1. Introduction

Transcatheter aortic valve replacement (TAVR) is the preferred treatment for many patients with aortic stenosis at increased surgical risk. However, neurologic complications remain a concern, particularly with regard to repositioning of the prosthetic valve during the procedure, which has been associated with early stroke [1, 2]. To mitigate this risk, transcatheter filters have been used to capture debris embolized during the TAVR procedure [3–5]. In the randomized SENTINEL trial, use of a double-filter cerebral embolic protection (CEP) device was associated with a trend towards a lower periprocedural stroke rate within 72 hours compared with patients undergoing unprotected TAVR [3]. Likewise, a large patient-level meta-analysis of CEP use in TAVR patients showed a significant reduction of periprocedural stroke and the composite of periprocedural mortality and stroke in patients in whom a double-filter CEP device was used [6].

Here, we evaluate the impact of selective use of an embolic protection device and of repositioning of the mechanically-expanded Lotus Valve in the RESPOND study, a large “all-comers” postmarket registry.

## 2. Methods

The RESPOND (Repositionable Lotus Valve System–Post-Market Evaluation of Real World Clinical Outcomes) study is a prospective, open-label, postmarket registry that enrolled 1014 patients with symptomatic aortic stenosis and elevated surgical risk at 41 centers in Europe, New Zealand, and Latin America [7].

The protocol was approved by the locally-appointed institutional review boards/ethics committees; the study was conducted in accordance with the International Conference on Harmonization Guidelines for Good Clinical Practice and the ethical principles outlined in the Declaration of Helsinki. All patients gave written informed consent. The study was sponsored by Boston Scientific Corporation and registered with ClinicalTrials.gov (NCT#02031302). The data and study protocol for this clinical trial may be made available to other researchers in accordance with Boston Scientific's Data Sharing Policy (http://www.bostonscientific.com/en-US/data-sharing-requests.html).

The Lotus Valve (Boston Scientific Corporation, Marlborough, MA) is a bioprosthetic aortic valve comprised of a braided nitinol wire frame with three bovine pericardial leaflets premounted on a preshaped delivery catheter and deployed via controlled mechanical expansion [8–10]. The Lotus Valve functions early in the deployment process, and rapid pacing is not required. A polymer membrane surrounding the lower half of the Lotus Valve was designed to reduce paravalvular regurgitation by filling the space between the native annulus and the prosthetic valve frame. Repositioning or retrieval of the valve is possible at any point prior to uncoupling and release. RESPOND evaluated the Lotus Valve sizes of 23 mm, 25 mm, and 27 mm, for implantation in native annulus sizes ≥20 mm to ≤27 mm.

The use of a CEP device in conjunction with the Lotus Valve was at the operators' discretion (i.e., selective use). All cases used the dual-filter Sentinel system (Claret Medical, a subsidiary of Boston Scientific Corporation, Marlborough, MA). Device specifics and mode of operations have been described elsewhere [11]. In brief, the device consists of proximal and distal nitinol filters with 140 *μ*m pores, deployed with a dedicated delivery catheter into the brachiocephalic trunk and the left common carotid, respectively.

The primary endpoint of RESPOND was all-cause mortality at 30 days and 1 year after procedure [7, 12]. Additional Valve Academic Research Consortium (VARC)-2 efficacy and safety outcomes [13] were evaluated, with all study end point-related clinical events (i.e., all-cause mortality and stroke events) reported by study investigators assessed by an independent medical reviewer (IMR). As per VARC-2 criteria, a stroke was defined as an acute episode of focal or global neurological dysfunction caused by brain, spinal cord, or retinal vascular injury as a result of hemorrhage or infarction; a transient episode of focal neurological dysfunction lasting <24 hours caused by brain, spinal cord, or retinal ischemia, without acute infarction, was considered a transient ischemic attack (TIA). The RESPOND study protocol did not require confirmation of TIA by MRI or neurologist evaluation. This analysis of RESPOND focuses on acute neurologic events (stroke and TIA) occurring within 30 days of TAVR.

Baseline and procedural characteristics were compared for patients with and without CEP use and valve repositioning; 2-sided *p* values were derived from a chi-squared or Fisher's exact test for categorical variables. A multivariate regression analysis evaluated clinical, anatomic, electrocardiographic, and procedural characteristics as potential predictors of stroke; these factors were assessed by logistic regression with Wald's chi-squared test and expressed as odds ratios with 95% confidence intervals. Significance was defined as *p* < 0.05. No imputation of missing data was performed. Statistical analysis was performed using SAS software, version 9.2 or more (SAS Institute, Cary, North Carolina).

## 3. Results

The RESPOND study enrolled 1014 patients between May 2014 and February 2016; 996 patients were implanted with a Lotus Valve (mean age: 80.8 years, 50.8% female, STS score 6.0 ± 6.9). A cerebral embolic protection device was used in 92 patients (9.2%). The patients in whom CEP was used were significantly more likely to have a history of congestive heart failure (59.8% vs. 35.5%; *p* < 0.001) and prior MI (26.1% vs. 14.8%; *p* < 0.01) (Table 1). Additionally, porcelain aorta (8.7% vs. 3.9%; *p*=0.05) and severe aortic valve calcification (50.0% vs. 32.2%; *p* < 0.001) were more common in patients with CEP. Repositioning of the valve occurred in 31.4% of procedures (*n* = 313/996), with a similar frequency in patients with (33.7%; 31/92 patients) and without (31.2%; 282/904 patients) CEP use. Overall, baseline characteristics were comparable among patients with and without repositioning (Table 1).

The overall rate of post-TAVR acute neurological complications (i.e., stroke/TIA) was 3.0% at 72 hours and 3.9% through 30 days (Table 2). Patients in whom CEP was used had a numerically lower rate of neurological events at all the time points, although the difference did not reach the statistical significance. The use of CEP was associated with a 2.1% absolute reduction in the risk of neurological events within 72 hours (relative risk reduction: 65.6%; *p*=0.51) and a 1.9% absolute reduction in the 30-day risk of stroke/TIA (relative risk reduction: 46.3%; *p*=0.57); however, these reductions were not statistically significant. Valve repositioning did not affect the stroke rate at any time point (Table 2). The rate of stroke within 72 hours was not significantly different in patients with and without repositioning regardless of whether CEP was also used (Table 3).

**Figure 1:**
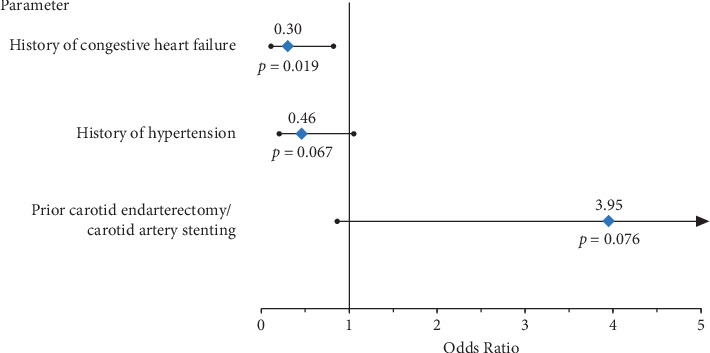
Multivariate regression analysis to evaluate potential predictors of acute stroke/TIA. Patients with a history of congestive heart failure were less likely to experience a stroke; however, multivariate modeling did not identify any additional factors that significantly predicted stroke/TIA through 72 hours.

A univariate analysis was performed to identify potential patient or procedural factors associated with acute stroke/TIA (≤72 hours after procedure) (Supplementary Table 1). Multivariate modeling revealed that patients with a history of congestive heart failure were less likely to experience a stroke (odds ratio [95% CI]: 0.30 [0.11, 0.82]; *p* = 0.02); however, the analysis did not identify any other factors that significantly predicted the stroke/TIA through 72 hours (Figure 1). A similar analysis for stroke/TIA at hospital discharge also did not identify any predictive factors (data not shown).

## 4. Discussion

Here, we present data from the multicenter, international RESPOND study, the largest study to date using the mechanically-expanded Lotus Valve in routine clinical practice. The study represents a selective use of dual-filter cerebral embolic protection, in 9.2% of patients. A 2.1% absolute reduction in the risk of periprocedural stroke was observed, corresponding to a two-thirds reduction in relative risk. This sizable numerical difference, however, failed to reach statistical significance due to a relatively small population of patients in which CEP was used. Repositioning of the prosthetic valve, which occurred in approximately one in three patients, was not associated with an increased risk of neurological events.

**Figure 2:**
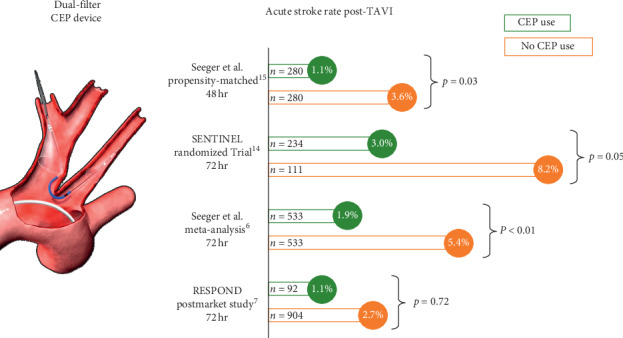
Comparative reduction in stroke risk with cerebral embolic protection. Use of a dual-filter cerebral embolic protection (CEP) device during transcatheter aortic valve replacement was associated with a numerical reduction in acute stroke in RESPOND, which is in concordance with that observed in other clinical analyses. ^15^Seeger et al., JACC: Cardiovascular Interventions, 10, 2297–2303, 2017. ^14^Data presented at SENTINEL CPS FDA Advisory Panel, February 23, 2017; accessed at https://www.fda.gov/media/103414/download. ^6^Seeger et al., European Heart Journal, 40, 1334–1340, 2019. ^7^Falk et al., European Heart Journal, 38, 3359–3366, 2017.

Although the reduction in stroke risk observed in RESPOND was not statistically significant, it is in concordance with the clinical results of other contemporary randomized trials and propensity score-matched analyses (Figure 2). The SENTINEL trial included 363 patients with a 2 : 1 randomization for CEP versus no CEP [3]. Within 72 hours, there was a strong trend towards stroke reduction with CEP compared with unprotected TAVR procedures (3.0% versus 8.2%, respectively; *p*=0.05) [14]. Likewise, in a large propensity score-matched population including 560 patients, use of an embolic protection device resulted in a significant reduction in stroke at 48 hours (1.1% vs. 3.6%; *p*=0.03), as well as within 7 days (1.4% vs. 4.6%; *p*=0.03) [15].

Although a recent meta-analysis of 16 TAVR studies performed with and without CEP could not confirm or exclude a difference in clinically-evident stroke (relative risk: 0.70; 95% CI: 0.38–1.29; *p* = 0.26) [16], a large patient-level meta-analysis combining data from 1306 patients drawn from the SENTINEL US IDE trial [3], CLEAN-TAVI study [4], and a large registry by Seeger et al. reconfirms the protective effect of use of CEP in TAVR patients [6]. The primary endpoint of the analysis was procedural stroke within 72 hours after TAVR according to VARC-2 criteria. The secondary endpoint was the combination of all-cause mortality or all-stroke within 72 hours after TAVR. In the propensity-matched population, which consisted of 533 patients treated with TAVR without CEP and 533 patients treated with TAVR with CEP, patients were similar with respect to baseline characteristics, procedural approach, and valve type. In patients undergoing TAVR with dual-filter CEP, procedural all-stroke was significantly lower compared with unprotected procedures (1.88% vs. 5.44%; odds ratio [95% CI]: 0.35 [0.17–0.72]; relative risk reduction: 65%; *p* = 0.003). In addition, all-cause mortality and all-stroke were significantly lower (2.06% vs 6.00%; odds ratio [95% CI]: 0.34 [0.17–0.68]; relative risk reduction: 66%; *p* = 0.001).

Imaging studies have increased the awareness of subclinical (silent) ischemic events occurring in TAVR patients, although the clinical impact of such events on cognitive function is unclear [17, 18]. The Neuro-TAVI study was a prospective, multicenter observational study designed to evaluate neurologic injury, cerebral ischemic lesion formation, and cognitive changes after TAVR [19]. The rate of disabling stroke at hospital discharge was 2.3%, similar to that observed in RESPOND. However, the study found that 1 in 5 patients exhibited clinically evident neurological impairment accompanied by imaging evidence of cerebral ischemia at discharge and that the effect was persistent, with 40% of patients exhibiting reduced cognitive measures 30 days after procedure. There is some evidence that use of CEP may help to mitigate the impact of subclinical ischemic events. An early randomized controlled trial of the Claret dual-filter device (MISTRAL-C) enrolling 65 TAVR patients found neurocognitive deterioration was present in 4% of patients with protection versus 27% of control patients (*p* = 0.017) at 30 days [5].

In this study, patients in whom CEP was used were more likely to have highly calcified aorta or aortic valve leaflets and thus had the potential for an increased risk of stroke related to dislodgement of debris during the procedure. Nevertheless, the rate of cerebral ischemic events was lower in this high-risk population with CEP compared with patients undergoing TAVR without cerebral protection. Calcification was not a significant predictor of stroke nor was the repositioning of the Lotus Valve associated with an increased risk of neurological events. Other studies of patients treated with the Lotus Valve have likewise shown that repositioning is not associated with an increased risk of major adverse cardiovascular or cerebrovascular events within 30 days [20, 21]. A comparative histopathological and histomorphometric analyses of captured debris by a double-filter embolic protection device demonstrated the total area of captured debris and the particle size captured varied depending on the type of TAVR device used (i.e., self-expanding vs. balloon-expandable vs. mechanically expanded) [22]; particles measuring larger than 1 mm were captured significantly more often with the balloon-expandable valve, while particle size was lowest with the mechanically-expanded Lotus Valve. Our findings support the use of the repositioning feature of the Lotus Valve without an increase in the risk of acute neurological complications.

Of note, our multivariate analysis in this large patient population did not identify an individual predictor for neurological events. It is challenging to predict the risk of stroke in a single patient undergoing TAVR, as there are several potential contributing factors which may be associated with the occurrence of stroke, including age, calcification of the valve and/or left ventricular outflow tract, atrial fibrillation, and presence of aortal plaques or calcification. Additionally, as the use of CEP in the study was at the operators' discretion (i.e., not randomized), there is the potential for bias related to patient selection. The results of the multivariate analysis should thus be interpreted with care and be considered to be hypothesis-generating.

This analysis has several other limitations. RESPOND is not a randomized study and thus lacks a direct comparator. The use of CEP in RESPOND was approximately 10%, which represents selective use of an embolic protection device in routine clinical practice, but nonetheless limits the sample size for analyses. Given the limited sample size and low overall incidence of stroke in the study, the multivariate analysis was underpowered and there were no observable trends related to potential predictors of stroke. Similarly, the sample size is too small to determine if there is a significant interaction between use of CEP, repositioning, and stroke.

The RESPOND protocol did not require a neurologic exam by a neurology professional; stroke events were site-reported and adjudicated by an independent medical reviewer, which may have contributed to a lower reported stroke rate in RESPOND compared with other major TAVR trials. Similarly, TIA was site-reported and thus may also have been underidentified. However, RESPOND stroke rates are consistent with data from the Society of Thoracic Surgeons/American College of Cardiology Transcatheter Valve Therapy (STS/ACC TVT) Registry, in which all site-reported stroke/TIA events were adjudicated by a board-certified cardiologist (in-hospital stroke rate: 2.0%; 30-day stroke rate: 2.8%). [23] Finally, patients in RESPOND were not evaluated for subclinical (silent) ischemic lesions or neurocognitive deterioration, thus the potential effect of such events in the study population is unknown.

The currently enrolling PROTECTED TAVR study (Stroke PROTECTion with SEntinel during Transcatheter Aortic Valve Replacement; NCT04149535) compares the 72-hour stroke rate in patients undergoing TAVR with or without the Sentinel dual-filter cerebral protection system. This large study (initial enrolment is set at 3000 patients) will incorporate a formal neurology consult in all patients where stroke is suspected and should provide important insights into the impact of CEP on the risk of periprocedural stroke.

## 5. Conclusions

Results from RESPOND suggest that the selective use of a CEP device for TAVR patients treated with the mechanically expanded Lotus was associated with a nonsignificant lower risk for stroke within 72 hours. The use of the repositioning feature of the Lotus Valve did not increase the stroke risk. In future studies, particularly as TAVR is extended to a broader population, it will be important to evaluate the cognitive function through a more formal neurological assessment.

## Figures and Tables

**Table 1:** Baseline characteristics in the RESPOND patient population, according to cerebral embolic protection use and valve repositioning.

Baseline characteristic	Embolic protection use			Valve repositioning		
	No CEP	CEP	*p* value	No REPOS.	REPOS.	*p* value
	*N* = 904	*N* = 92		*N* = 683	*N* = 313	
Age (years)	80.9 ± 6.5	80.0 ± 6.4	0.24	80.9 ± 6.3	80.5 ± 7.0	0.39
Gender (female)	49.9%	59.8%	0.07	52.0%	48.2%	0.27
BMI (kg/m^2^)	27.3 ± 5.6	27.1 ± 5.0	0.84	27.4 ± 5.7	26.9 ± 5.1	0.22
STS score (%)	6.0 ± 7.0	5.8 ± 5.8	0.89	5.6 ± 6.0	6.8 ± 8.6	0.02
EuroSCORE 2011 (%)	8.1 ± 8.4	7.1 ± 8.2	0.28	8.0 ± 8.2	8.0 ± 8.8	0.98
Diabetes, medically treated	22.3%	23.1%	0.87	22.7%	21.7%	0.73
History of hyperlipidemia	54.4%	57.8%	0.54	54.5%	55.2%	0.82
History of hypertension	78.4%	86.8%	0.06	79.5%	78.5%	0.72
Porcelain aorta	3.9%	8.7%	0.05	4.4%	4.2%	0.86
Aortic valve calcification, severe (site-reported)	3.2.%	50.0%	<0.001	32.7%	36.4%	0.24
Hostile chest	0.8%	3.3%	0.06	0.9%	1.3%	0.52
History of cerebrovascular accident	9.2%	12.0%	0.39	8.5%	11.5%	0.13
History of coronary artery disease	55.9%	57.6%	0.76	56.7%	54.8%	0.58
History of congestive heart failure	35.5%	59.8%	<0.001	36.3%	40.8%	0.17
History of atrial fibrillation	34.5%	27.8%	0.20	34.5%	32.6%	0.55
Prior MI	14.8%	26.1%	<0.01	15.3%	17.2%	0.46
Prior PCI	29.5%	33.0%	0.49	29.6%	30.3%	0.81
Prior CABG	11.7%	18.5%	0.06	11.1%	15.0%	0.08
Prior pacemaker	13.7%	8.7%	0.18	13.3%	13.1%	0.92

**Table 2:** Neurological complications through 30 days after TAVR in patients with and without CEP use and valve repositioning.

Post-TAVR neurological complications	Overall population	Embolic protection use			Valve repositioning		
		No CEP	CEP	*p* value	No REPOS.	REPOS.	*p* value
	*N* = 996	*N* = 904	*N* = 92		*N* = 683	*N* = 313	
≤72 hr							
Stroke/TIA	3.0% (30)	3.2% (29)	1.1% (1)	0.51	3.1% (21)	2.9% (9)	0.86
Stroke	2.5% (25)	2.7% (24)	1.1% (1)	0.72	2.5% (17)	2.6% (8)	0.95
Disabling stroke	1.8% (18)	2.0% (18)	0.0% (0)	0.40	1.6% (11)	2.2% (7)	0.49
Discharge							
Stroke/TIA	3.7% (37)	3.9% (35)	2.2% (2)	0.57	3.8% (26)	3.5% (11)	0.82
Stroke	3.1% (31)	3.3% (30)	1.1% (1)	0.35	3.1% (21)	3.2% (10)	0.92
Disabling stroke	2.2% (22)	2.4% (22)	0.0% (0)	0.25	1.9% (13)	2.9% (9)	0.33
30-days							
Stroke/TIA	3.9% (39)	4.1% (37)	2.2% (2)	0.57	3.8% (26)	4.2% (13)	0.79
Stroke	3.2% (32)	3.4% (31)	1.1% (1)	0.35	3.1% (21)	3.5% (11)	0.71
Disabling stroke	2.3% (23)	2.5% (23)	0.0% (0)	0.26	1.9% (13)	3.2% (10)	0.21

**Table 3:** Effect of valve repositioning and cerebral embolic protection use on acute stroke.

Stroke ≤72 hr after TAVR	Valve repositioning	No repositioning	*p* value
	*N* = 313	*N* = 683	
With CEP	*n* = 31	*n* = 61	1.00
All-stroke	0.0% (0)	1.7% (1)	
No CEP	*n* = 282	*n* = 622	0.81
All-stroke	2.8% (8)	2.6% (16)	

## Data Availability

The data and study protocol for this clinical trial may be made available to other researchers in accordance with the Boston Scientific Data Sharing Policy (http://www.bostonscientific.com/enUS/data-sharing-requests.html).

## Abbreviations

BMI: Body mass index
CABG: Coronary artery bypass graft
CEPP: Cerebral embolic protection
COPD: Chronic obstructive pulmonary disease
EOA: Effective orifice area
MI: Myocardial infarction
PCI: Percutaneous coronary intervention
STS: Society of Thoracic Surgeons
TAVR: Transcatheter aortic valve replacement
TIA: Transient ischemic attack
VARC: Valve Academic Research Consortium.

## Supplementary Materials

Supplementary Materials Supplementary Table 1: univariate analysis for multivariate modeling of stroke/TIA ≤ 72 hours after procedure.
