# Supplementary material for: Insights on Embolic Protection, Repositioning, and Stroke: A Subanalysis of the RESPOND Study
Source: J Interv Cardiol. 2020 May 17;2020:3070427. doi: 10.1155/2020/3070427 (PMC7251468; doi:10.1155/2020/3070427)
Supplement: Supplementary Materials — Supplementary Table 1: univariate analysis for multivariate modeling of stroke/TIA ≤ 72 hours after procedure. [file 3070427.f1.pdf]

Supplementary Table 1. Univariate analysis for multivariate modeling of stroke/TIA ≤72 hours post-procedure

Variable	N	Events	Odds Ratio 95% C.I.	P value
Univariate analysis				
History of congestive heart failure	989	30	0.40[0.16,1.00]	0.049
History of hypertension	988	29	0.49[0.22,1.07]	0.072
History of myocardial infarction	989	30	0.18[0.02,1.31]	0.090
Prior carotid endarterectomy/carotid artery stenting	972	29	3.42[0.76,15.37]	0.109
Pre-procedure aortic valve area (Effective Orifice Area by echo)	875	26	0.20[0.02,1.57]	0.124
History of hyperlipidemia requiring medication	978	27	0.56[0.26,1.22]	0.145
History of cerebrovascular accidents	993	30	1.96[0.73,5.26]	0.179
History of chronic obstructive pulmonary disease (COPD)	994	30	0.38[0.09,1.62]	0.191
History of transient ischemic attacks	989	30	1.98[0.67,5.85]	0.214
Embolic protection device	996	30	0.32[0.04,2.40]	0.270
Minimum diameter of the right iliofemoral system	734	19	0.86[0.64,1.13]	0.278
History of Coronary Artery Disease (CAD)	995	30	0.68[0.33,1.40]	0.294
History of Coronary Artery Bypass Graft (CABG) surgery	996	30	0.50[0.12,2.12]	0.346
Prior balloon aortic valvuloplasty	989	30	1.69[0.39,7.33]	0.487
Minimum diameter of the left iliofemoral system	727	18	0.92[0.71,1.18]	0.504
Male gender	996	30	0.78[0.38,1.63]	0.515
NYHA Class IV	925	25	0.54[0.07,4.02]	0.544

Pre-procedure Left Ventricular Ejection Fraction (LVEF)	903	28	1.01[0.98,1.04]	0.571
Prior pacemaker implant	996	30	0.72[0.22,2.41]	0.596
RCA	861	21	1.03[0.91,1.16]	0.611
History of percutaneous coronary intervention	993	30	1.18[0.55,2.56]	0.669
Current anginal status - CCS3	996	30	1.37[0.32,5.90]	0.676
Aortic valve calcification - Moderate	925	25	0.84[0.36,1.97]	0.689
Weight	989	30	1.00[0.97,1.02]	0.691
Height	987	30	0.99[0.96,1.03]	0.691
Overstretch ≥10% per LVOT Area	578	12	0.80[0.25,2.56]	0.708
STS Score	849	28	1.01[0.96,1.06]	0.725
Current anginal status - CCS2	996	30	0.81[0.24,2.70]	0.727
EuroSCORE (2011)	925	27	1.01[0.96,1.05]	0.739
History of Atrial Fibrillation	985	30	1.13[0.53,2.41]	0.746
Overstretch - per LVOT Area (%)	578	12	1.00[0.97,1.04]	0.784
Porcelain aorta	991	30	0.75[0.10,5.67]	0.785
Mean aortic pressure gradient (mm Hg)	906	27	1.00[0.98,1.02]	0.834
History of dialysis dependent renal failure	993	30	1.24[0.16,9.47]	0.834
Age at time of consent	996	30	1.01[0.95,1.06]	0.837
Overstretch ≥10% per LVOT Diameter	535	12	1.13[0.34,3.82]	0.839
Overstretch - per LVOT Diameter (%)	535	12	1.01[0.93,1.09]	0.863
NYHA Class III	925	25	1.07[0.47,2.46]	0.865
Aortic Regurgitation	887	24	0.92[0.36,2.36]	0.866
Current anginal status - None	996	30	1.07[0.47,2.44]	0.868
Aortic valve calcification - Mild	925	25	0.92[0.31,2.70]	0.873
Medically treated Diabetes	996	30	1.06[0.45,2.51]	0.889
NYHA Class II	925	25	1.06[0.44,2.58]	0.892

BMI (kg/m^2)	986	30	1.00[0.92,1.07]	0.907
NYHA Class I	925	25	1.09[0.14,8.34]	0.930
Height of LCA	865	22	1.00[0.87,1.14]	0.960
Aortic valve calcification - Severe	925	25	0.98[0.43,2.24]	0.964
Current anginal status - CCS1	996	30	0.97[0.29,3.27]	0.965
History of Atrial Flutter	987	30	NE	0.978
COPD - Supplemental oxygen dependent	994	30	NE	0.983
Severe liver disease/cirrhosis	993	30	NE	0.985
Hostile chest/unfavorable chest wall anatomy	995	30	NE	0.987
Current anginal status - CCS4	996	30	NE	0.989
Multivariate analysis[*]				
History of Congestive Heart Failure	959	28	0.30[0.11,0.82]	0.019
History of hypertension			0.46[0.20,1.05]	0.067
Prior carotid endarterectomy/carotid artery Stenting			3.95[0.86,18.09]	0.076

[*]Factors from the univariate model with $P \leq 0.20$ (shaded boxes) were modeled using a stepwise multivariate procedure in a logistic regression model. The significance level thresholds for entry and exit of independent variables into the multivariate model were set at 0.1.
NE, Not Evaluable
